# Direct observation of mineral–organic composite formation reveals occlusion mechanism

**DOI:** 10.1038/ncomms10187

**Published:** 2016-01-06

**Authors:** Kang Rae Cho, Yi-Yeoun Kim, Pengcheng Yang, Wei Cai, Haihua Pan, Alexander N. Kulak, Jolene L. Lau, Prashant Kulshreshtha, Steven P. Armes, Fiona C. Meldrum, James J. De Yoreo

**Affiliations:** 1 The Molecular Foundry, Lawrence Berkeley National Laboratory, Berkeley, California 94720, USA; 2 Bioscience and Biotechnology Division, Physical and Life Sciences Directorate, Lawrence Livermore National Laboratory, Livermore, California 94550, USA; 3 School of Chemistry, University of Leeds, Leeds LS2 9JT, UK; 4 Department of Chemistry, University of Sheffield, Brook Hill, Sheffield S3 7HF, UK; 5 Department of Mechanical Engineering, Stanford University, Stanford, California 94305, USA; 6 Department of Chemistry, Zhejiang University, Hangzhou 310027, China; 7 Physical Sciences Division, Pacific Northwest National Laboratory, Richland, Washington 99352, USA

## Abstract

Manipulation of inorganic materials with organic macromolecules enables organisms to create biominerals such as bones and seashells, where occlusion of biomacromolecules within individual crystals generates superior mechanical properties. Current understanding of this process largely comes from studying the entrapment of micron-size particles in cooling melts. Here, by investigating micelle incorporation in calcite with atomic force microscopy and micromechanical simulations, we show that different mechanisms govern nanoscale occlusion. By simultaneously visualizing the micelles and propagating step edges, we demonstrate that the micelles experience significant compression during occlusion, which is accompanied by cavity formation. This generates local lattice strain, leading to enhanced mechanical properties. These results give new insight into the formation of occlusions in natural and synthetic crystals, and will facilitate the synthesis of multifunctional nanocomposite crystals.

## Abstract (web summary)

The occlusion of biomacromolecules can endow biominerals with enhanced mechanical properties. Here, the authors use *in situ* atomic force microscopy and micromechanical simulations to trace micelle incorporation in calcite to shed light on the mechanism of occlusion and cavity formation.

## Graphical abstract

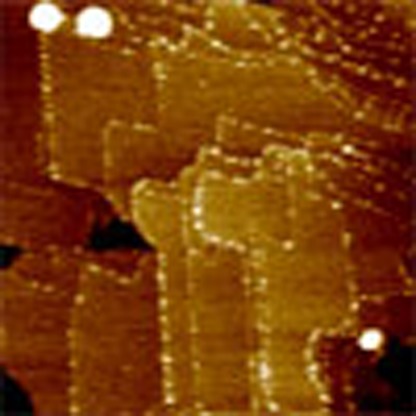

Soluble additives are widely used to control crystallization processes, providing an experimentally simple and versatile strategy to generate crystals with defined polymorphs, morphologies and sizes^1^. Significant insights into additive-directed crystallization, and the effects of these additives on crystal properties, have come from *in vitro* studies of biomineralization processes^2^,^3^, where the use of advanced characterization methods have demonstrated that the macromolecules active in controlling crystallization become occluded within the crystal structure, modifying crystal texture and lattice strain^4^,^5^,^6^,^7^,^8^, causing local disorder^9^ and enhancing mechanical properties^10^. Tomography-based studies have even succeeded in revealing atomic-scale structural^11^ and chemical^12^ characteristics of interfaces between the crystal lattice and occluded organic materials.

Translation of this strategy to synthetic systems promises the ability to control crystal properties and to generate composite structures^5^,^13^,^14^,^15^,^16^. However, implementation of this approach is currently limited by a poor understanding of the mechanisms by which nanoscale additives are incorporated within single crystals. Much of our knowledge comes from *in situ* atomic force microscopy (AFM) studies supported by computational models, in which changes in crystal shape and step speed (growth rate) are used as indirect probes of additive/crystal interactions^17^. Without the ability to image the individual additives, this process cannot be fully characterized or understood. In this study, we profit from recent demonstrations that high densities of functionalized nanoparticles can be occluded within inorganic crystals^5^,^15^,^16^ to reveal the mechanisms that govern the adsorption and occlusion of organic additives within single calcite crystals.

## Results

### Adsorption of copolymer micelles during calcite growth

**Figure 1:**
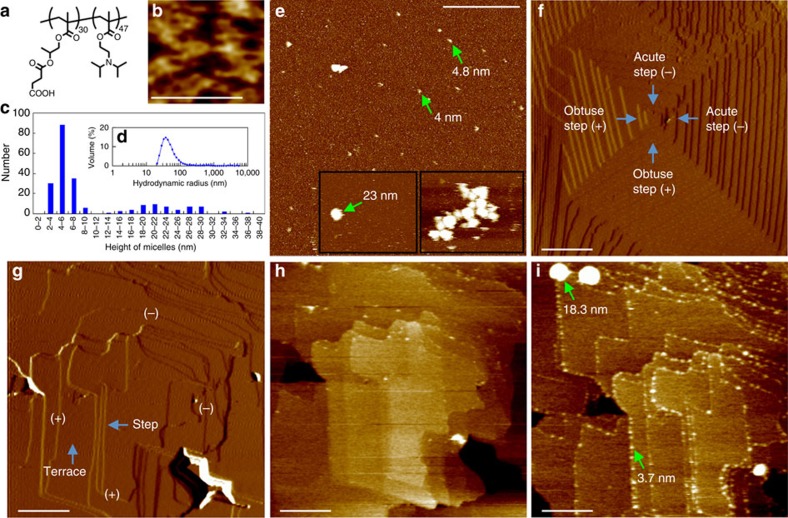
Structure and adsorption dynamics of carboxylated micelles on calcite. (**a**) Chemical structure and (**b**) morphologies of copolymers observed on positive (poly-L-lysine-treated) mica surface in water. (**c**) Height distribution measured by AFM and (**d**) hydrodynamic radii measured by DLS of micelles in calcium carbonate solution (*σ*=∼1.81, pH=∼8.5). The heights of the micelles were determined from images such as (**e**), which is a positive mica surface showing micelles belonging to two populations, one ∼3.5–6.5 nm and another ∼20–30 nm in height (left inset). Some aggregates were also observed (right inset). The main image and insets are at the same scale. (**f**) Morphology of {104} calcite growth surface showing growth hillocks composed of two crystallographically distinct types of atomic steps (acute (−) and obtuse (+)) generated at screw dislocations^18^. (**g**) Calcite surface in (near)—equilibrium solution (step speed=0). (**h**) Same surface as in **g** after exposure for ∼2 min to a micelle-containing solution at near-equilibrium conditions (step speed=0), demonstrating that micelles were immobilized at the step edges rather than on the terraces, with slightly greater affinity for the acute steps over obtuse steps. (**i**) Same surface as in **h** after exposure for another 48 min to flowing micelle-containing solution showing overwhelming dominance of steps edge adsorption and that micelles adsorbed on calcite formed ellipsoids with the short axis perpendicular to the plane of the crystal face. Scale bars, (**b**) 50 nm, (**e**) 300 nm, (**f**) 600 nm and (**g**–**i**) 300 nm. All images were collected in tapping mode except for **f** and **g** which collected in contact mode.

Our approach employed carboxylated (Fig. 1a,b) and sulfonated (Supplementary Figs 1 and 2) block copolymer micelles as additives that act as mimics of the biomacromolecules occluded within biominerals. Incorporation of these micelles within calcite generates ‘artificial biominerals' with microstructures and mechanical properties comparable to those of biogenic calcite^5^. Crucially, they are sufficiently large enough to be visualized simultaneously with atomic steps on crystal surfaces using *in situ* AFM.

Characterization of the carboxylated micelles in bulk CaCO_3_ solution using dynamic light scattering (DLS) revealed a monomodal population with an average diameter of ∼75 nm (Fig. 1d), while two distinct populations were observed on mica (Fig. 1c,e) and calcite surfaces, together with occasional micellar aggregates (Fig. 1e, right inset). The adsorbed micelles on mica were ellipsoidal in morphology, exhibiting heights and diameters of 23.1 (average)±4.6 nm (s.d.) and 41.9±5.8 nm for the large micelles and 5.2±1.4 and 15.6±3.8 nm for the small ones, respectively. The calcite surfaces in pure CaCO_3_ solutions consisted of terraces separated by 0.31 nm high ‘acute' and ‘obtuse' atomic steps^18^ emanating from screw dislocations (Fig. 1f,g). Under continuous flow of micelle-containing solution at or near equilibrium with respect to calcite (Fig. 1g), both small and large micelles were immobilized at the step edges rather than on the terraces (Fig. 1h,i). Adsorption was complete within a few minutes and adsorption was slightly preferred on the acute over the obtuse steps (63±3.1% versus 37±2.6%) (Fig. 1h and Supplementary Movie 1). Identical behaviour was observed at all flow rates (including zero) and for all supersaturations investigated, indicating that neither binding to terraces nor crystallization speed played a direct role in micelle incorporation.

### Incorporation of the micelles into calcite single crystals

**Figure 2:**
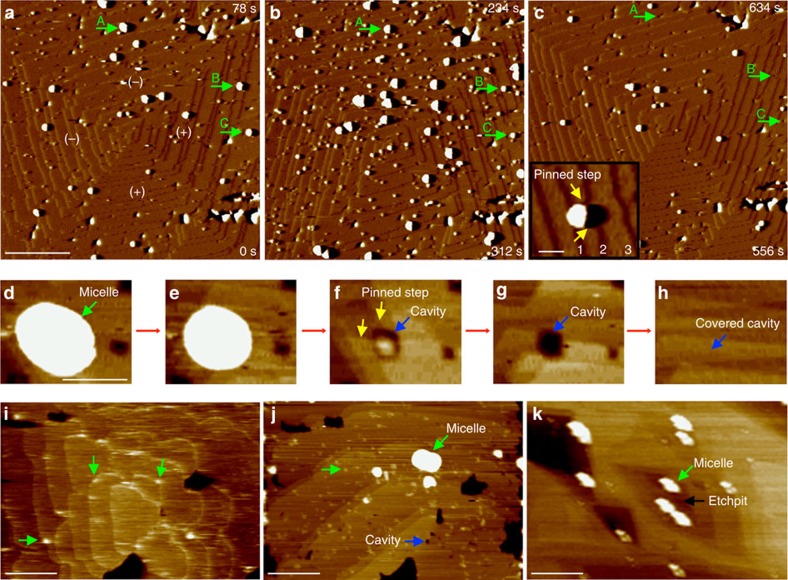
Incorporation of carboxylated micelles and associated cavity generation. (**a**–**c**) Sequential *in situ* 3 × 3 μm AFM images of growing calcite surface at *σ*=1.49. Particles indicated by arrows in (**a**) subsequently show a decrease in height (**b**), before undergoing complete burial (**c**). The inset in **c** shows one step (1) that has just reached a micelle, one (2) that has just closed around it and another (3) that has nearly recovered to a straight morphology with little overall inhibition. The times (*t*) at which the bottom and top of the images were collected are given in lower and upper right corners of images, respectively, where the bottom of (**a**) was arbitrarily set to *t*=0 s. The copolymer concentration was (**a**,**b**) 127 nM and (**c**) zero. (**d**–**h**) Detailed view of the incorporation of a large micelle (*σ*=2.66). (**d**; 0 s) An ellipsoidal micelle with a height of 19.6 nm and in-plane diameters of 93 nm is adsorbed to an acute step (see Supplementary Fig. 5f for determination of true micelle diameter). (**e**; 34 s) As incorporation proceeds, the micelle decreases in height (13.3 nm) and its shape evolves to that of an ellipsoid with a circular cross-section and reduced diameter of 84 nm. (**f**; 2 min 35 s) Upon further burial, a gap (blue arrow) forms around the micelle with the diameter further reduced to 33 nm (height: 0.8 nm). (**g**; 3 min 10 s) The cavity remains (blue arrow) after complete micelle burial before eventually closing over, (**h**; 31 min 58 s). (**i**) Calcite surface with adsorbed micelles (green arrows) in micelle-containing solution at (near-) equilibrium (step speed=0, copolymer concentration: 69 nM). (**j**) Upon introduction of highly supersaturated solution (*σ*=2.66) and consequent step advancement, many micelles (green arrows) appear because of increased capture rate by steps. Many have already become incorporated and formed cavities (blue arrow). (**k**) Reappearance of incorporated micelles in etchpits upon dissolution. Scale bars; (**a**) 750 nm, (**d**, inset to **c**) 100 nm and (**i**–**k**) 300 nm. Images **a**–**h** and **i**–**k** were collected in contact and tapping mode, respectively.

Micelle incorporation was then studied over a range of supersaturations (*σ*=∼ 0–2.66; see the Methods section for definition of *σ*). Both the micelle diameter and height decreased with the passage of each step as the micelles were gradually entrapped (Fig. 2a–c) (Supplementary Fig. 3a–c and Supplementary Movie 2). As incorporation continued, a cavity began to form around the perimeter of each micelle and persisted—often for significant time periods—after complete burial of the micelle (A–C in Fig. 2c,d–g). Cavities associated with large micelles had greater diameters and persisted for much longer times than those of small ones (compare Fig. 2g,h with Supplementary Fig. 4b,c), but all were eventually either covered or possibly filled in by advancing steps (Fig. 2h). Significantly, cavities also appeared on dissolution of the composite crystals in undersaturated solutions. Dissolution first generated etchpits and then cavities, and ultimately exposed the buried micelles (Fig. 2k and Supplementary Fig. 4d–i).

Higher supersaturations led to a greater number density of incorporated micelles because the density of steps generated at a dislocation source—and therefore the probability of capturing a micelle from solution—increases quadratically with supersaturation^18^ (Fig. 2i,j). Close examination of the micelle-step boundary also showed that steps experienced little or no inhibition by adsorbed micelles (inset, Fig. 2c and Supplementary Fig. 3d–i), as demonstrated by their similar growth rates and straight edges in both pure and micelle-containing solutions (compare Fig. 2a–c with Fig. 1f). This behaviour contrasts with that of other macromolecular adsorbates that bind to kink sites at steps and effectively block addition of solute ions at similar additive concentrations and solution supersaturations^19^, presumably because the effective copolymer concentration is greatly reduced by micelle formation.

**Figure 3:**
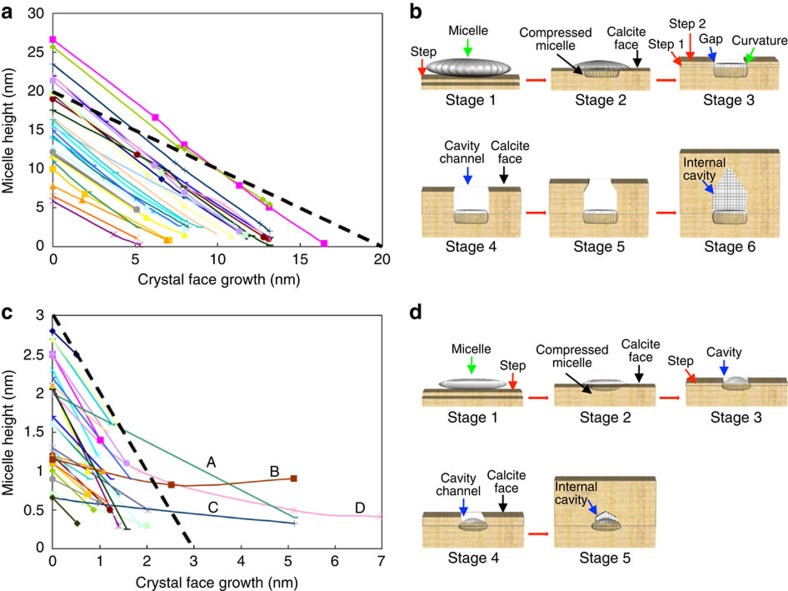
Micelle height trajectories and schematic of micelle incorporation. (**a**) Plot showing changes in heights of individual large micelles relative to crystal face versus absolute height of the growing crystal face. (Crystal face growth (nm)=0 marks time when each micelle is first imaged and end point of each trajectory marks last micelle image before complete incorporation.) Errors in height caused by tip compression of the micelles under these conditions were <3 nm for the largest micelles and <0.5 nm for the smallest at crystal face growth=0, leading to maximum errors in slope of 8–11 %. (**b**) Schematic diagram illustrating the incorporation of the large micelles. A micelle adsorbs to a step as an ellipsoid (stage 1) and contracts vertically, while it is compressed laterally (stage 2) as the calcite face advances. A gap then begins to form around the micelle periphery (stage 3). Successive steps do not advance beyond this gap, creating a cavity. As growth continues, the gap develops into a cylindrical channel (stage 4), whose width decreases (stage 5) until it closes to form an internal cavity (stage 6). (**c**) Plot of height changes for small micelles determined as in **a** showing shallower slopes than for large micelles, including slopes > −1 (for example, A–D). (**d**) Schematic representation showing that the incorporation process of small micelles is similar to that of large micelles, except that micelles elongate vertically between stages 2 and 3 and channel lengths is much smaller. Face growth rate=0.033 nm s^−1^ (*σ*=1.49).

Careful measurements of micelle heights revealed a further key aspect of incorporation, which is best illustrated by comparing the relative heights of individual large micelles above the calcite surface to the absolute position of the growing crystal face (Fig. 3a). Here, a trajectory parallel to the thick dashed line, which has a slope of unity, represents a micelle that is simply buried by the growing face without any change in its absolute height. Our data show that the actual micelle trajectories had slopes <−1, which demonstrates that the micelles continuously retracted vertically during burial (Supplementary Fig. 5e for detailed analysis). At the same time, the micelles also underwent simultaneous lateral compression (Fig. 2d,e), suggesting loss of associated water molecules and/or collapse of polymer chains during occlusion. In all cases, the ellipsoidal shapes of the large micelles were maintained during crystal growth (Fig. 3b), with the micelle diameter and height decreasing from 94.1±9.4 and 19.9±3.1 nm upon adsorption to 38.2±5.9 and 13.1±1.9 nm after burial, respectively.

The small micelles with initial heights of about 3.2±1.1 nm, exhibited similar behaviour with one distinct difference; slopes were initially slightly <−1, but often became >−1 during the last 2 nm of burial (Fig. 3c). These micelles therefore retracted downward slightly during the initial phase of compression, but once buried roughly halfway, they began to extend upward with continued growth. Based on the analysis from Fig. 3c and AFM images, the diameters and heights of the small micelles were estimated to change from 32.0±6.0 and 3.2±1.1 nm, respectively, upon adsorption to 9.0±3.1 and 4.6±0.7 nm, respectively, after burial.

### Micromechanical simulations of micelle incorporation

**Figure 4:**
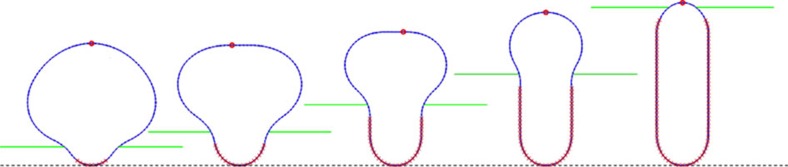
Micromechanical simulation of spherical micelle incorporation. During burial, lateral micelle compression is accompanied by an initial downward contraction, followed by upward extension. The bottom dashed line indicates the surface on which the micelle is initially attached, while the green solid lines represent the top surface of the crystal. The red crosses mark the fixed portion of the (buried) micelle. The red circle marks the highest point of the micelle. While the true initial micelle shape is that of a flattened ellipsoid rather than a sphere, the mechanical response to lateral compression resembles that observed in Fig. 3d.

Simulations of micelle incorporation into a crystal growing through layer-by-layer addition were also performed to understand the morphological behaviour observed for the small micelles, as well as the mechanical consequences of burial. For simplicity, we assumed that the micelles were initially spherical and used a two-dimensional model, in which the initial micelle shape is represented as a circle (Fig. 4). (Note that while this simple model correctly explains the general pattern of shape change of the small micelles, it predicts a higher final aspect ratio than observed experimentally, because the actual initial micelle shape is a flattened ellipsoid (Fig. 3d, stage 1)). The bottom of the circle was fixed to the initial crystal surface to mimic binding to the step edge. As the height of the crystal was increased, it was allowed to compress the micelles laterally until the pressure exerted by the crystal was balanced by the bending resistance of the micelle. Some representative results are given in Supplementary Figs 6–8.

Two conditions were considered for the boundary between the crystal and the buried part of the micelle. In the first, the buried boundary was allowed to continuously relax, thereby mimicking continued communication with the solution. Under this condition the crystal exerts an isotropic pressure field on the micelle. Thus, the buried micelle returns either to a spherical shape (under low pressure) or to some non-convex but symmetrical shape (under high pressure). Hence, the final shape of the micelle cannot be ellipsoidal, because the bending energy of the micelle can be reduced without changing the work done by the pressure field if the micelle is transformed to a sphere of the same volume. In the second condition, the buried boundary was assumed to be static, which simulates no ion exchange with the solution. The crystal-micelle boundary therefore becomes fixed shortly after burial beneath the top surface of the crystal. This model predicts that the top of the micelle retracts vertically at the beginning of incorporation, before eventually extending upwards, as observed experimentally for small micelles.

## Discussion

Based on our experimental data, micromechanical simulations and previous transmission electron microscopy (TEM) data, which show that the occluded micelles—and any associated cavities that may be retained—are ellipsoidal in form, we can now reconstruct the entire incorporation process for both large (Fig. 3b) and small micelles (Fig. 3d). Following micelle binding to step edges (Fig. 3b,d, stage 1), the passing steps begin to compress the micelles (Fig. 3b,d, stage 2). For each layer, the steps stop when the increase in bending energy of the micelle balances the chemical potential that drives step advancement. The bending resistance of the micelle thus strains the surrounding crystal, which reduces the driving force for crystallization around the micelle. This then contributes to the formation of a gap (Fig. 3b,d, stage 3) in a manner similar to the creation of hollow cores in the strain field of a dislocation^20^. As the micelle becomes approximately half buried, the difficulty of extending the crystal steps over the surface of the underlying micelle—and thus creating an overhang—may also contribute to gap formation. As the growth front passes beyond the micelle, the gap develops into a channel (Fig. 3b,d, stage 4), which ultimately closes over as the strain field of the compressed micelle recedes behind the advancing growth front (Fig. 3b,d, stages 5 and 6).

These data do not enable us to determine whether cavities are retained or eliminated during the growth process. Either scenario should be possible depending on the supersaturation and particle size. A simple extrapolation to biominerals—in which similar sized occlusions are observed by TEM—suggests they may be a combination of cavities and proteins. Indeed, this is consistent with a previous TEM study of the aragonite plates in nacre^21^, and would also explain why occlusions can far exceed the sizes expected for individual proteins^22^. However, previous studies of a wide range of calcite/particle composite crystals using scanning electron microscopy and TEM have not reported evidence for retained cavities^14^,^16^. This is particularly clear for occluded gold nanoparticles, which should be easily distinguished from an associated cavity using TEM by virtue of their high electron density. The cavities observed upon dissolution of the composite crystals suggest that the cavities may remain; however, these might also be attributable to the high lattice strain present in the vicinity of the micelles, which renders these regions more soluble than strain-free regions (Fig. 2k and Supplementary Fig. 4d–i).

This study provides significant new insights into additive-controlled crystallization processes. Considering first the calcite/micelle system, the previous study of Kim *et al*.^5^ concluded from a purely structural analysis that the ellipsoidal occlusions seen in TEM corresponded to occluded micelles that were incorporated as a result of their affinity for adsorption to the crystal face with a morphology determined by that interaction. The mechanistic understanding obtained from our current *in situ* observations refines this picture. Micelles actually bind to advancing steps and undergo a morphological change during occlusion as a result of compression by the steps. Kim *et al*.^5^ also showed that the calcite/micelle crystals are harder than pure calcite, where this change was associated with a large compressive strain gradient in the crystal. The current work implies this local strain is a direct result of the chemical driving force behind the step advancement, which ensures that the steps will continue to compress the micelle until the resulting lattice strain energy equals the solution chemical potential.

Much of our current understanding of additive occlusion derives from optical and theoretical studies on incorporation of large particulates within crystals during their freezing transitions^23^,^24^. Under those conditions, particle entrapment occurs only above a critical growth velocity of the solid–liquid interface^23^,^24^, and depends on many factors including the thermal diffusivity of the particle and melt, wetting of the crystal face by the particles, the size and buoyancy of the particles, and the melt viscosity. Particle entrapment during growth from solution, in contrast, has received considerably less attention^14^, although it has been suggested that similar factors may be important.

The model that emerges here stands in stark contrast to those previously proposed^23^,^24^,^25^, in which particles are incorporated either because of the slow diffusive velocity of the liquid towards the crystal surface relative to the interface growth velocity or because of hydrodynamic forces that correlate with crystallization speed. Instead the particles become entrapped because they bind specifically to steps, enabling successive steps to close around them. Further, the increase in incorporation efficiency at higher growth rates is a consequence of the greater rate of step generation with increasing supersaturation^18^, rather than the enhanced growth front velocity. These results therefore provide a new understanding of the dynamics of additive/crystal interactions. Combined with the insight they provide concerning evidence on the strain-induced toughening of minerals, these findings will inform the synthesis of novel composite crystals through the optimization of variables, such as adsorbate–step interactions, bending rigidity and supersaturation.

## Methods

### Micelle stock solution preparation

PSPMA_30_-PDPA_47_ block copolymer powder was dissolved in MilliQ water for which the pH was initially adjusted to ∼4.8 with 1 M HCl solution. The prepared stock solution volumes were typically 100 ml and included 5.76 μM (fixed concentration) copolymer. (See Supplementary Information of ref. 5 for the copolymer synthesis.) After the copolymers were completely dissolved, rapid micelle formation was then induced by increasing the solution pH to ∼9.5–∼10 by adding 1 M NaOH solution. The pH of the stored stock solutions decreased over time from the initial value to ∼8.0. However, the micelles kept their micellar structure, because the pK_a_ values^26^ of PSPMA and PDPA are ∼5.5 and 6.3, respectively. Micelle stock solutions of sulfonated SBA: PHPMA_30_-PDPA_47_ copolymer were prepared in the same manner (see Supplementary Methods and Supplementary Fig. 2 for the synthesis and characterization of the sulfonated copolymer).

### Micelle-containing calcium carbonate solution preparation

Calcium carbonate (CaCO_3_) solutions (volume 100 ml) at supersaturations (*σ*)=∼0–2.66 with a calcium to typical carbonate ratio of ∼0.5 were prepared. Here, *σ* is defined by *σ*=ln *a*(Ca^2+^) *a*(CO_3_^2−^)/*K*_sp_ where *a*(Ca^2+^) and *a*(CO_3_^2−^) are the activities of the calcium and carbonate ions, respectively, and *K*_sp_ is the solubility constant for calcite. The activities of calcium and carbonate ions were obtained using the multicomponent speciation program Visual Minteq^27^, which uses the Davies equation to give the ion activity coefficient. The desired amounts of micelle stock solution (typically <2.2 ml) were first introduced into ∼46 ml CaCl_2_.2H_2_O (reagent grade) solutions at pH ∼10 (adjusted using NaOH). The solution volume was then adjusted to 50 ml using MilliQ water. These solutions were mixed with 50 ml NaHCO_3_ (reagent grade) solutions containing the desired amounts of NaCl to give CaCO_3_ solutions with ionic strength (I) of ∼0.05 M. In this way, micelle-containing CaCO_3_ solutions with a typical copolymer concentration <127 nM and *I*=∼0.05 at pH ∼8.5 were prepared.

### *In situ* AFM imaging

Freshly cleaved geologic calcite (Iceland spar, Ward's Natural Science, Rochester, NY) was glued to the AFM specimen disk to expose a fresh {104} surface for investigation. A commercial fluid cell (MTFML, Veeco Probes) with O-ring was placed on the cleaved calcite face inside an AFM. (Multimode Nanoscope IIIa or VIII from Digital Instruments, Santa Barbara, CA). A syringe pump was used to flow solution through the fluid cell and imaging was performed at room temperature using commercially available SiN cantilevers (Bruker, NP-S with spring constant of 0.12 N m^−1^ for imaging on calcite surface, and Olympus, TR400PSA with spring constant of 0.08 N m^−1^ for imaging on mica surface) with a nominal radius of 15 nm (as reported by the manufacturer).

The *in situ* images of the calcite surface were obtained in either contact mode or tapping mode at solution flow rates of 0.3 ml min^−1^. Images were typically collected at a scan rate 3.3 Hz (lines per second) with 256 scan lines per image. We found that applying the minimum possible scan force, a suitable scan rate, and scan size were critical to being able to record quality, reproducible images of the micelles on the calcite surface. Tapping mode images were collected at ≥ 60% of cantilever free amplitude. The above scan parameters were appropriate for imaging areas between 1 × 1 μm and 3 × 3 μm and for obtaining reasonable acquisition rates. To account for the distortion in step orientations because of finite scan rates and non-zero step speeds^28^ in images collected *in situ*, step speeds were obtained by orienting the fast scan direction perpendicular to true step directions and measuring the change in the apparent angle of the steps in the images collected during upward and downward scans^28^. To image the negatively charged micelles on mica surfaces, the surface charge of the mica was adjusted from negative to positive by depositing poly-L-lysine on freshly cleaved mica before introducing the micelle solutions (∼50 μl) into the fluid cell.

### Dynamic light scattering

Dynamic light scattering measurements of carboxylated PSPMA_30_-PDPA_47_ micelles were obtained on a Malvern Zetasizer Nano Series ZS (Malvern Instruments) and analysed with the provided Zetasizer software. The samples came from the same calcium carbonate solution as those used for AFM imaging. Measurements were recorded at 25 °C, the refractive index of the buffer was estimated to be 1.330 and the viscosity 0.8894 cP.

### Determination of micelle and step heights

Heights of micelles and steps were obtained by analysing height profiles from images such as those in Fig. 1e,i using standard Veeco Nanoscope image analysis software. In addition, the scanning probe image processor (SPIP 5.1.4) was used for measuring heights of micelles from images obtained on mica surfaces. The height distribution of micelles in Fig. 1c was based on measurements from three 1 × 1 μm *in situ* images, including Fig. 1e.

### Simulations of micelle incorporation

A two-dimensional model was developed in which the shape of the micelle was represented as a continuous and closed line (that is, loop). The line is discretized into a set of nodes {**r**_i_}, where **r**_i_=(*x*_*i*_, *y*_*i*_), *i*=1, …, *N*. The nodes are connected to their neighbours: **r**_*i*_ is connected to **r**_*i*+1_ for *i*=1,…, *N*-1 and **r**_*N*_ is connected to **r**_*1*_. A total free energy *F* is defined as a function of nodal positions.


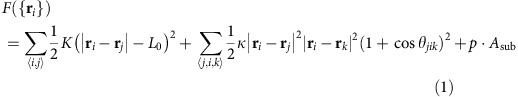


where *K* is the stretching stiffness and *L*_0_ is the equilibrium ‘bond length' between neighbouring nodes, *κ* is the bending stiffness and *θ*_*jik*_ is the angle formed between ‘bonds' *i-j* and *i-k*, *p* is the pressure exerted by the crystal and *A*_sub_ is the part area enclosed by the loop below the top surface of the crystal. The three terms in the free energy function *F* represent the contributions from the stretching of the membrane at the surface, the bending of the membrane and the interaction with the crystal, respectively. The free energy contribution from the volume change of the micelle (that is, the bulk contribution) is not included in the above expression. We have assumed the stretching stiffness to be sufficiently large so that the perimeter of the micelle remains essentially constant during the deformation. Therefore, the shape change of the micelle is mainly determined by the competition between the bending energy (second term) and the interaction with the crystal (third term). In an extended model, we have also considered the effect of the bulk free energy contribution due to the volume change of the micelle (Supplementary Note 1). However, we found that if the bulk contribution is too large, the micelle shape remains essentially circular throughout the entire burial process, which is inconsistent with the experimental observation. Deformation of the micelle is only obtained when the bulk term is sufficiently small, but in this case the predicted behaviour is qualitatively the same as that for the bulk term equal to zero. Therefore, we have excluded the bulk term in the above free energy expression.

For a given position of the top surface of the crystal, *y*_sub_, the nodes evolve in the steepest direction, that is


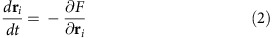


until equilibrium positions are reached. *y*_sub_ is then incremented by a small step and the nodes are allowed to evolve into their new equilibrium positions. The simulation shown in Fig. 4 was performed using the following (dimensionless) parameters: *K*=2, *L*_0_=0.63, *κ*=1, *P*=10^−4^. Nodes with *y*_*i*_ < *y*_sub_—2 are fixed (red crosses in Fig. 4) to simulate the condition of no communication between the solution and the buried part of the micelle.

## Additional information

**How to cite this article:** Cho, K. R. *et al*. Direct observation of mineral-organic composite formation reveals occlusion mechanism. *Nat. Commun.* 7:10187 doi: 10.1038/ncomms10187 (2016).

## Supplementary Material

Supplementary Information Supplementary Figures 1-8, Supplementary Notes 1-2, Supplementary Methods and Supplementary References

Supplementary Movie 1 Adsorption dynamics of carboxylated PSPMA_30_-PDPA_47_ micelles onto calcite surface (1.5 × 1.5 μm) shown in Fig. 1g-i of the main paper. This movie shows the adsorption dynamics of the carboxylated micelles for 63 minutes and 12 seconds in equilibrium condition. Because calcite steps did not move in this condition, the adsorption site of the micelles could be identified. The micelles adsorbed directly to step edges rather than terraces, and adsorption to acute over obtuse steps was slightly preferred.

Supplementary Movie 2 Incorporation dynamics of carboxylated PSPMA_30_-PDPA_47_ micelles into calcite single crystal. This movie shows the incorporation dynamics of the carboxylated micelles, which occurred in the area inside the red box (1 × 1 μm) shown in Supplementary Fig. 3a for 32 minutes 31seconds. Steps advanced past micelles, which adsorbed to them with little or no inhibition, and the continual passage of steps encapsulated the micelles into the crystal. For the explanation of step morphologies shown in this movie, see Supplementary Fig.3a-c legend and the In situ AFM imaging part of the Methods section.

Supplementary Movie 3 Adsorption dynamics of sulfonated SBA: PHPMA_30_-PDPA_47_ micelles onto calcite surface (1.5 × 1.5 μm) shown in Supplementary Fig. 1a,b. This movie shows adsorption dynamics of the sulfonated micelles for 105 minutes and 19 seconds in equilibrium condition. Because steps did not move in this condition, the adsorption site of the micelles could be identified. As with the carboxylated PSPMA_30_-PDPA_47_ micelles, these micelles adsorbed directly to step edges rather than terraces. However, in contrast to the carboxylated micelles, adsorption to acute was strongly preferred over adsorption to obtuse steps.

Supplementary Movie 4 Incorporation dynamics of sulfonated SBA: PHPMA_30_-PDPA_47_ micelles into calcite single crystal and the effect of supersaturation on their adsorption and incorporation. This movie shows adsorption and incorporation events of the sulfonated micelles on the calcite surface (1.5 × 1.5 μm) shown in Supplementary Fig. 1, which occurred for 27 minutes and 11 seconds. When solution supersaturation increased, more micelles adsorbed to steps and were incorporated by the growing steps (micelle capture enabled accurate imaging of morphology).

## References

[b1] Song R.-Q. & Cölfen H. Additive controlled crystallization. CrystEngComm 13, 1249–1276 (2011).

[b2] Weiner S. & Addadi L. Design strategies in mineralized biological materials. J. Mater. Chem. 7, 689–702 (1997).

[b3] Meldrum F. C. & Colfen H. Controlling mineral morphologies and structures in biological and synthetic systems. Chem. Rev 108, 4332–4432 (2008). 19006397 10.1021/cr8002856

[b4] Berman A. . Intercalation of sea-urchin proteins in calcite: study of a crystalline composite material. Science 250, 664–667 (1990). 17810868 10.1126/science.250.4981.664

[b5] Kim Y. Y. . An artificial biomineral formed by incorporation of copolymer micelles in calcite crystals. Nat. Mater. 10, 890–896 (2011). 21892179 10.1038/nmat3103

[b6] Berman A., Addadi L. & Weiner S. Interactions of sea-urchin skeleton macromolecules with growing calcite crystals—a study of intracrystalline proteins. Nature 331, 546–548 (1988).

[b7] Pokroy B., Fitch A. N. & Zolotoyabko E. The microstructure of biogenic calcite: a view by high-resolution synchrotron powder diffraction. Adv. Mater. 18, 2363–2368 (2006).

[b8] Kim Y. Y. . A critical analysis of calcium carbonate mesocrystals. Nat. Commun. 5, 4341 (2014). 25014563 10.1038/ncomms5341 PMC4104461

[b9] Metzler R. A. . Nacre protein fragment templates lamellar aragonite growth. J. Am. Chem. Soc. 132, 6329–6334 (2010). 20397648 10.1021/ja909735y

[b10] Weiner S., Addadi L. & Wagner H. D. Materials design in biology. Mater. Sci. Eng. C 11, 1–8 (2000). 10.1023/a:1008989719560 15348099

[b11] Li H., Xin H. L., Muller D. A. & Estroff L. A. Visualizing the 3D internal structure of calcite single crystals grown in agarose hydrogels. Science 326, 1244–1247 (2009). 19965470 10.1126/science.1178583

[b12] Gordon L. M. & Joester D. Nanoscale chemical tomography of buried organic-inorganic interfaces in the chiton tooth. Nature 469, 194–197 (2011). 21228873 10.1038/nature09686

[b13] Brif A., Ankonina G., Drathen C. & Pokroy B. Bio-inspired band gap engineering of zinc oxide by intracrystalline incorporation of amino acids. Adv. Mater. 26, 477–481 (2014). 24174394 10.1002/adma.201303596

[b14] Kim Y.-Y. . Bio-inspired synthesis and mechanical properties of calcite-polymer particle composites. Adv. Mater. 22, 2082–2086 (2010). 20544895 10.1002/adma.200903743

[b15] Kulak A. N. . One-pot synthesis of an inorganic heterostructure: uniform occlusion of magnetite nanoparticles within calcite single crystals. Chem. Sci 5, 738–743 (2014).

[b16] Kulak A. N., Yang P. C., Kim Y. Y., Armes S. P. & Meldrum F. C. Colouring crystals with inorganic nanoparticles. Chem. Commun. 50, 67–69 (2014). 10.1039/c3cc47904h 24202647

[b17] De Yoreo J. J., Wierzbicki A. & Dove P. M. New insights into mechanisms of biomolecular control on growth of inorganic crystals. CrystEngComm 9, 1144–1152 (2007).

[b18] Teng H. H., Dove P. M. & Orme C. A. Thermodynamics of calcite growth: baseline for understanding biomineral formation. Science 282, 724–727 (1998). 9784126 10.1126/science.282.5389.724

[b19] Elhadj S., De Yoreo J. J., Hoyer J. R. & Dove P. M. Role of molecular charge and hydrophilicity in regulating the kinetics of crystal growth. Proc. Natl Acad. Sci. USA 103, 19237–19242 (2006). 17158220 10.1073/pnas.0605748103 PMC1748210

[b20] De Yoreo J. J., Land T. A. & Lee J. D. Limits on surface vicinality and growth rate due to hollow dislocation cores on KDP {101}. Phys. Rev. Lett. 78, 4462–4465 (1997).

[b21] Gries K., Kroger R., Kubel C., Fritz M. & Rosenauer A. Investigations of voids in the aragonite platelets of nacre. Acta Biomater. 5, 3038–3044 (2009). 19427933 10.1016/j.actbio.2009.04.017

[b22] Li H. . Calcite prisms from mollusk shells (Atrina Rigida): swiss-cheese-like organic-inorganic single-crystal composites. Adv. Funct. Mater. 21, 2028–2034 (2011).

[b23] Uhlmann D. R., Chalmers B. & Jackson K. A. Interaction between particles and a solid-liquid interface. J. Appl. Phys. 35, 2986–2993 (1964).

[b24] Rempel A. W. & Worster M. G. The interaction between a particle and an advancing solidification front. J. Cryst. Growth 205, 427–440 (1999).

[b25] Li H. Y. & Estroff L. A. Calcite growth in hydrogels: assessing the mechanism of polymer-network incorporation into single crystals. Adv. Mater. 21, 470–473 (2009).

[b26] Vo C. D., Armes S. P., Randall D. P., Sakai K. & Biggs S. Synthesis of zwitterionic diblock copolymers without protecting group chemistry. Macromolecules 40, 157–167 (2007).

[b27] Gustaffson J. P. Visual Minteq 2.30 edn. http://vminteq.lwr.kth.se/ (2004).

[b28] Land T. A., De Yoreo J. J. & Lee J. D. An *in-situ* AFM investigation of canavalin crystallization kinetics. Surf. Sci. 384, 136–155 (1997).

