# Supplementary material for: Direct observation of mineral–organic composite formation reveals occlusion mechanism
Source: Nat Commun. 2016 Jan 6;7:10187. doi: 10.1038/ncomms10187 (PMC4729825; doi:10.1038/ncomms10187)
Supplement: Supplementary Information — Supplementary Figures 1-8, Supplementary Notes 1-2, Supplementary Methods and Supplementary References [file ncomms10187-s1.pdf]

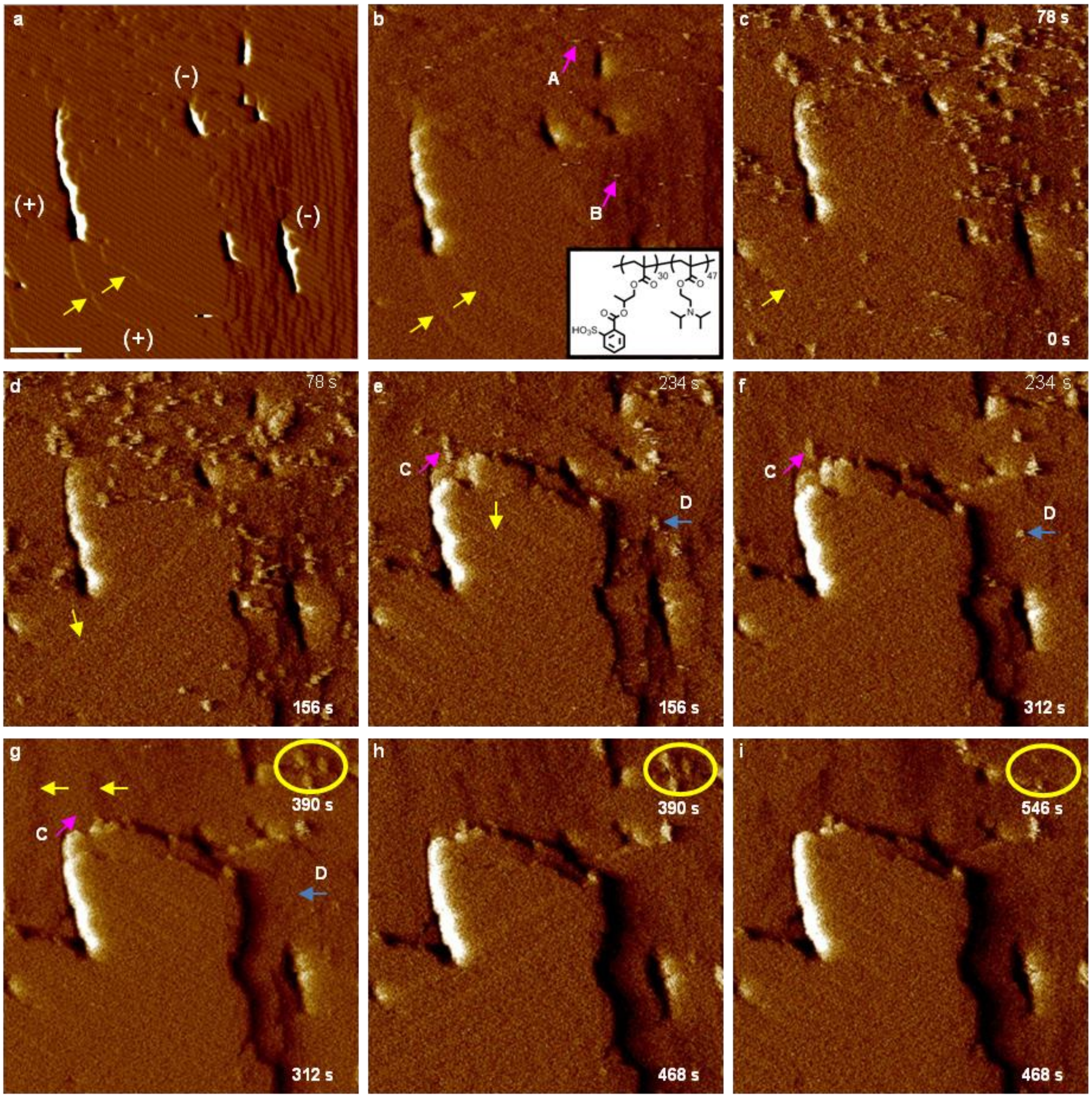

Supplementary Figure 1. Adsorption and incorporation processes of sulfonated block copolymer SBA: $PHPMA_{30}$-$PDPA_{47}$ micelles into calcite single crystals. (**a**) Calcite growth surface in (near)-equilibrium calcium carbonate solution (step speed = 0) showing atomic steps (acute (-) and obtuse (+)) generated at a screw dislocation. (**b**) Adsorption of the sulfonated micelles from the micelle-containing (near)-equilibrium calcium carbonate solution to growth surface shown in **a** (Inset to **b** is chemical structure of the sulfonated block copolymer and the copolymer concentration in the solution was 69 nM). Due to equilibrium state of solution, steps in **b** are immobile, as shown by comparison of steps indicated by yellow arrows in **b** and those in **a**. Micelles such as A and B indicated by pink arrows adsorbed directly to acute step edges with little or no adsorption to either obtuse steps or terraces, resulting in highly inhomogeneous micelle distribution. (Note that **a** was obtained using contact mode AFM imaging, which generally gives higher lateral resolution on the mineral surfaces than the tapping mode. However, micelles were swept away by the AFM tip in the contact mode, so the tapping mode

was used for **b** through **i**.) Micelles in **b** are distorted from their true morphology due to their movement during scanning of the tip even using the minimum tapping force that allowed for image collection. (**c and d**) Effect of sudden increase in supersaturation on micelle adsorption and capture. When highly supersaturated micelle-free calcium carbonate solution (σ = 2.66) arrived at the crystal surface, the resulting step advancement (noted by a yellow arrow showing different step location and configuration in **b**) led to capture of micelles. Micelle capture enabled accurate imaging of morphology (compare morphologies in **b** and those in **c** and **d**). Note that as also seen when comparing **b** with **c** and **d**, a larger number of micelles adsorbed to steps when supersaturation increased. Like the case of carboxylated $PSPMA_{30}$-$PDPA_{47}$ micelles, this was mainly due to the increased step density at higher supersaturation. Starting in **c**, images (**c-i**) were collected consecutively every 78 seconds while the micelle-free calcium carbonate solution was flowing continuously. Times (t) at which the bottom and top of the images were collected are given in lower and upper right corners of the images, respectively, where the bottom of **c** is arbitrarily set to t = 0 s. (**e-i**) Incorporation of adsorbed micelles. Many micelles shown in **d** were incorporated while steps were growing. Micelles (C, D) indicated by pink and blue arrows in **e** are seen in **f** and then not seen in **g** because they were incorporated into the growing calcite. Note that in **g**, the micelle (C) initially shown in **e** was incorporated by acute steps such as those indicated by two yellow arrows and which moved from left to right. Micelles in a yellow circle in **g** are incorporated (see yellow circles in **h** and **i**) by the same process seen for micelles (C, D). The change in apparent step orientation between images collected during upward and downward scans is clear from the steps indicated by the yellow arrows in **d** and **e**. (See the **In situ AFM imaging** part of the **Methods** section and ref. 1 for a detailed explanation of this effect.) See Supplementary Movies, 3 and 4 for the adsorption and incorporation dynamics of these sulfonated block copolymer micelles. (**a-i**) are 1.5×1.5 µm images and scale bar in **a** is 300 nm. All images were collected in tapping mode except **a**, which was collected in contact mode.

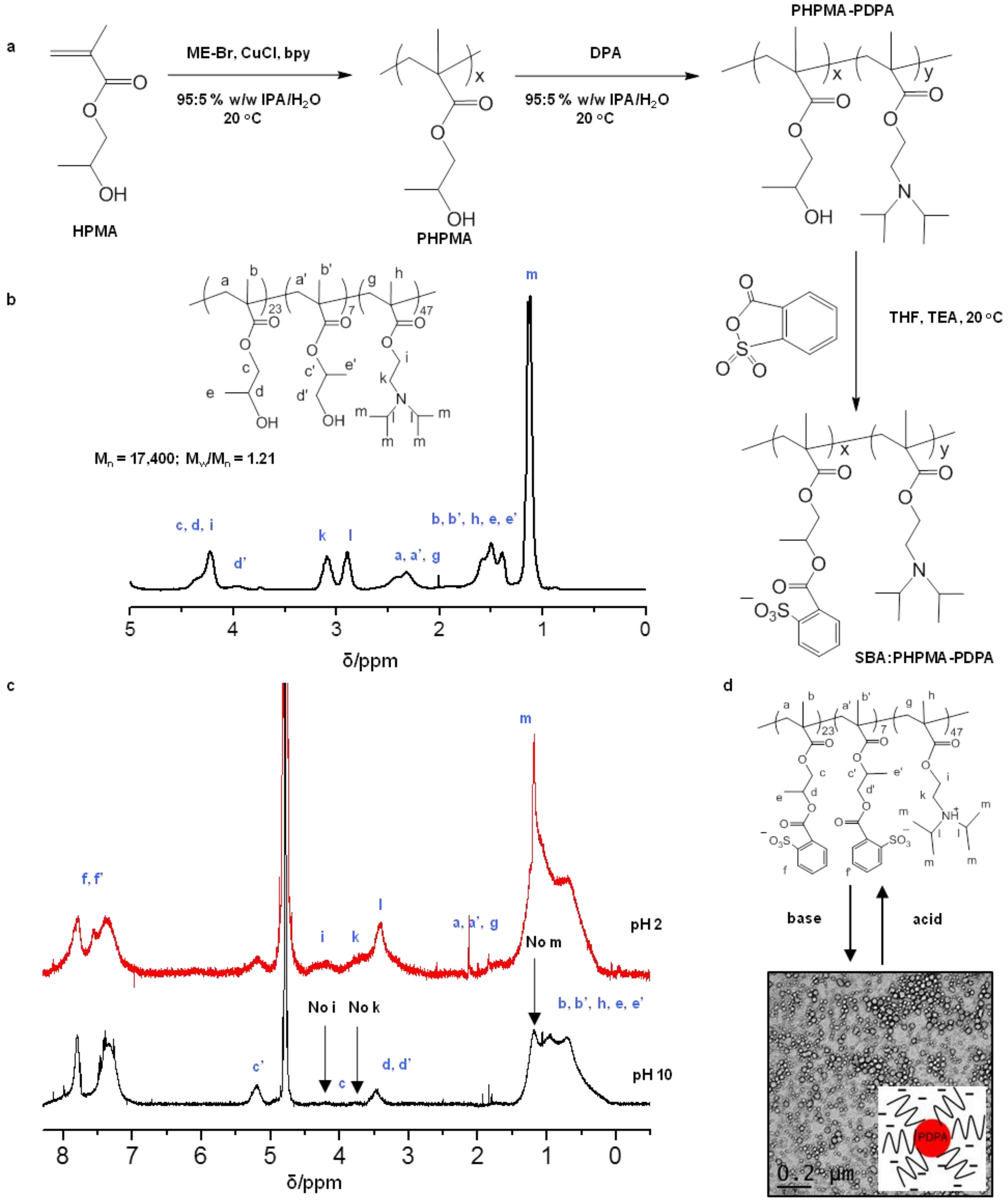

Supplementary Figure 2. Synthesis and characterization of zwitterionic diblock copolymer SBA:PHPMA$_{30}$-PDPA$_{47}$. (**a**) Reaction scheme for the synthesis of SBA:PHPMA$_{30}$-PDPA$_{47}$ zwitterionic diblock copolymer in a solution of 2-propanol (IPA) and water (IPA/water = 95/5 %

w/w) at 20 ^{o}C via atom transfer radical polymerization (ATRP) (Note that only the major isomer of 2-hydroxypropyl methacrylate (HPMA) monomer is shown here for clarity; ME-Br is 2-(4-morpholino)ethyl 2-bromoisobutyrate initiator; bpy is 2,2'-bipyridine; DPA is 2-(diisopropylamino)ethyl methacrylate; THF is tetrahydrofuran; TEA is triethylamine; SBA is 2-sulfobenzoic acid cyclic anhydride. See Supplementary Methods for more detailed explanation of the reaction). (**b**) THF gel permeation chromatography (GPC) molecular weight data and ^{1}H NMR spectrum (in d_5-pyridine) recorded for the $PHPMA_{30}$-$PDPA_{47}$ diblock copolymer precursor. (**c**) ^{1}H NMR spectra recorded for SBA:$PHPMA_{30}$-$PDPA_{47}$ diblock copolymer dissolved in D_2O in the presence of 0.10 M NaCl as a function of pH (adjusted using DCl or NaOD). At pH 2, the SBA:PHPMA and PDPA blocks are both charged and hence can dissolve molecularly in NaCl solution. Thus all signals due to these two solvated blocks are visible in the ^{1}H NMR spectrum. Increasing the solution pH to 10 using NaOD led to the disappearance of the PDPA signals at δ 1.2, δ 3.8, and δ 4.2, since the deprotonated PDPA block (pK_a ~6.3) becomes hydrophobic at this pH. The anionic SBA:PHPMA block acts as a stabilizer at high pH (its zeta potential is ~ -40 mV at pH 8). (**d**) TEM image of SBA:$PHPMA_{30}$-$PDPA_{47}$ diblock copolymer micelles dried from aqueous solution at pH 10.

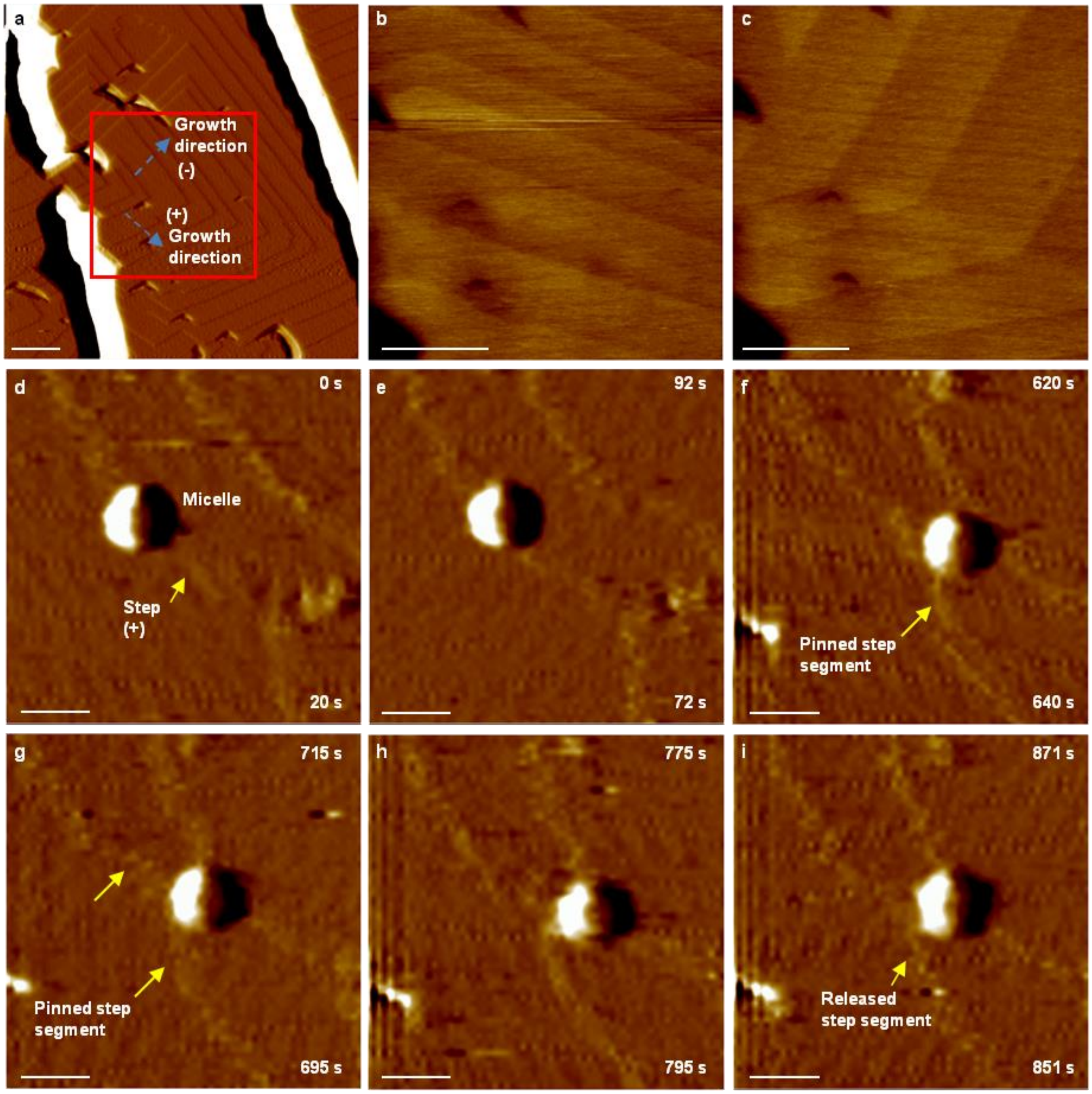

Supplementary Figure 3. Region of calcite surface seen in Supplementary Movie 2 and detailed view of step-micelle interaction during incorporation of a carboxylated $PSPMA_{30}$-$PDPA_{47}$ micelle. (**a**) The incorporation process shown in the Supplementary Movie 2 was observed in the area (1 × 1 µm) inside red box. Dotted arrows indicate the growth direction of acute (-) and obtuse (+) steps. The orientations of steps shown in **a** are close to their true orientations because of the relatively slow step growth speed compared to the scan rate[1] (scan rate: 5.09 lines s^{-1} with 256 scan lines per image, scan size: 2.18 × 2.18 µm). (**b** and **c**) Images collected during downward (**b**) and upward (**c**) scans and which appear in Supplementary Movie 2. Unlike the step orientations shown in **a**, step orientations seen in **b** and **c** differ from each other and are greatly distorted from their true morphologies, because, at this supersaturation (σ = ~1.81), steps moved rapidly relative to the scan rate (scan rate: 3.3 lines s^{-1} with 256 scan lines per image, scan size: 1 × 1 µm). Scale bars (**a-c**): 300 nm. (**d-i**) Consecutive images showing an obtuse (+) step moving from right to left (at 0.3 nm s^{-1}) passing by an adsorbed micelle at low

supersaturation (σ = ~0.05). Times (t) at which the top and bottom of the images were collected are marked at the right corners of the top and bottom of the images, where the top of **d** was arbitrarily set to t = 0 s. The step is passing by the micelle in **d** and **e**, but becomes pinned by the micelle as its leading edge advances beyond the micelle (yellow arrows and “Pinned step segment”) in **f** and **g**. The pinned step segments are eventually released as shown in **h** and **i** (“Released step segment” in **i**). Scale bars (**d-i**): 100 nm. All images were collected in contact mode except **b** and **c**, which was collected in tapping mode.

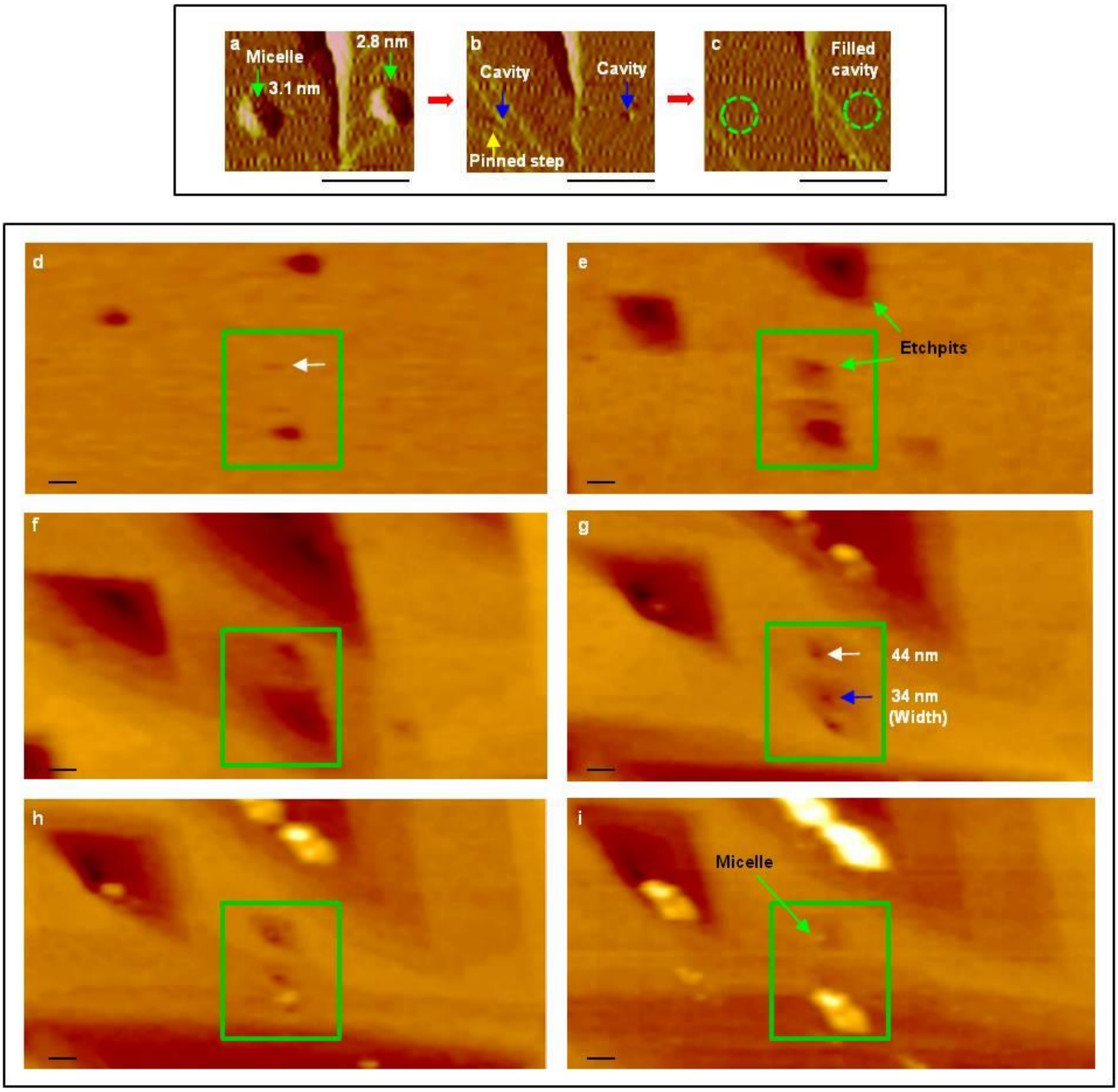

Supplementary Figure 4. Generation of cavity accompanied by incorporation of small micelle (a-c) and reappearance of the internal cavities and micelles during dissolution of the micelle-calcite composite crystal (d-i). (**a**) Small micelles with approximate true diameters of 36 nm (left) and of 29 nm (right) and heights of 3.1 nm (left) 2.8 nm (right) indicated by green arrows being incorporated during growth at σ = 2.66. (See Supplementary Fig. 5f and Supplementary Note 2 for calculation of true micelle diameter from AFM images.) (**b**) Micelles are completely incorporated leaving behind small cavities as indicated by blue arrows. (**c**) Cavities are filled in or closed over as indicated by the dotted green circles. Time t of image collection: **a** 0 sec (arbitrary), **b** 140 sec, and **c** 451 sec. Scale bars: 100 nm. (**d**) Growing calcite surface after incorporating micelles in supersaturated solution. White arrow indicates location of cavity that had been just closing over during growth. (**e-i**) Same surface during subsequent dissolution in under-saturated solution showing development of etchpits at the sites of the

cavities seen in **d** (e.g., see green arrows in **e**). Internal cavities with widths of 44 nm and 34 nm appear within these primary etchpits, as indicated, e.g., by the white and blue arrows in **g** (compare the location indicated by the white arrow in **d** and that in **g**). As the dissolution proceeds (**g-i**), numerous buried micelles are revealed in the growing etchpits. The green arrow in **i** shows the buried micelle appearing in the cavity with a width of 44 nm indicated by the white arrow in **g**. Scale bars (**d-i**): 50 nm. All images were collected in tapping mode except **a** – **c**, which were collected in contact mode.

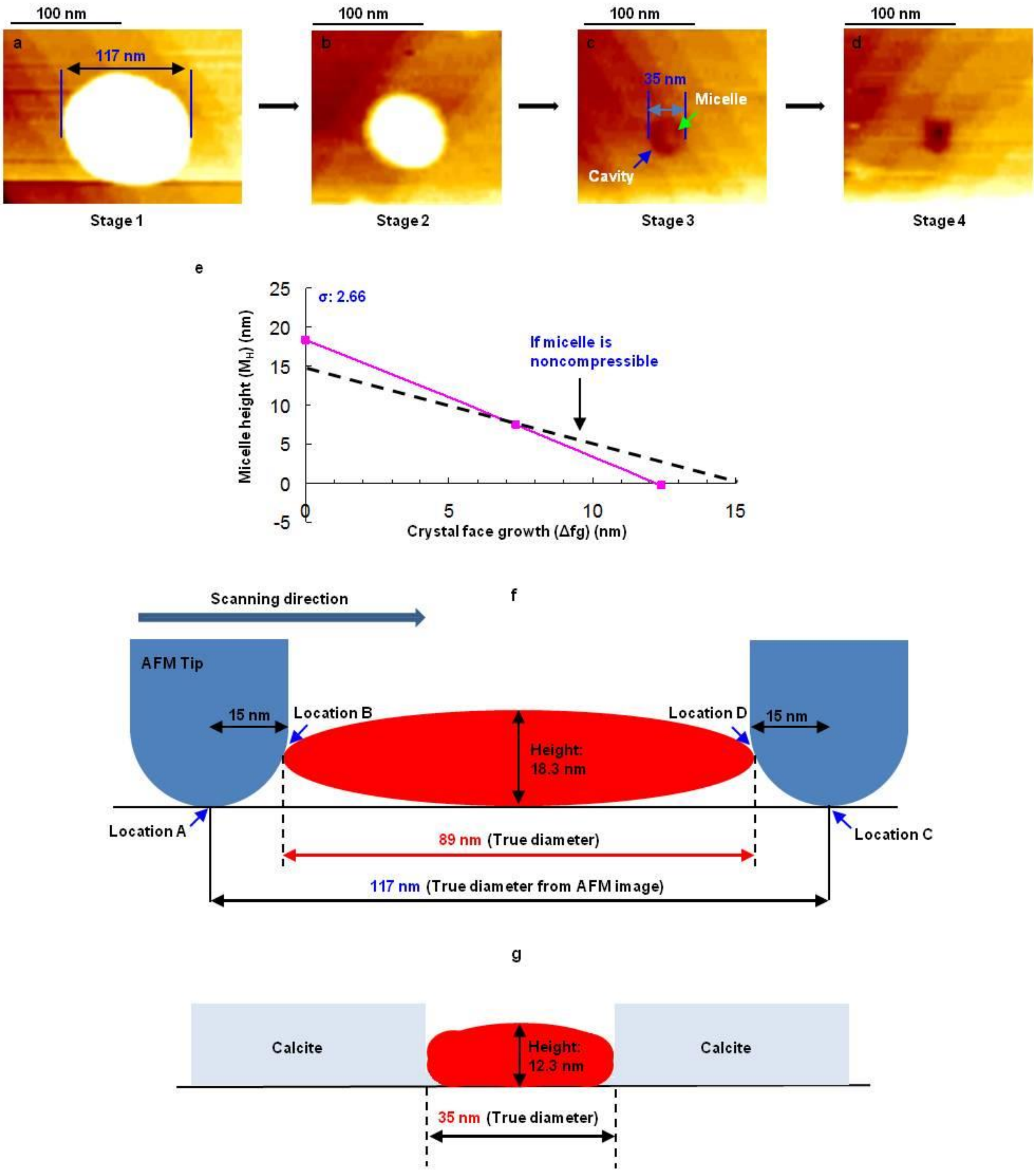

Supplementary Figure 5. Actual micelle shape upon adsorption to and shape evolution during incorporation. (**a-d**) Detailed view of sequential incorporation process of a large micelle (σ = 2.66, face growth rate: 0.08 nm s^{-1}). A large micelle just after adsorption (**a**) has a height of 18.3 nm and an apparent diameter of 117 nm, and is slightly ellipsoidal with about 15-20 % difference in diameter along the short and long axes. Once adjusted for tip convolution, the approximate true diameter is found to be 89 nm (for method of approximating true diameter, see

f, and Supplementary Note 2). Thus the ratio of height to diameter is 0.21, showing the micelle is an oblate ellipsoid. (**e**) Plot of micelle height (M_H) measured relative to the calcite surface in **a** (Stage 1), **b** (Stage 2) and **c** (Stage 3) versus calcite face growth (Δfg). As shown, the micelle with an initial height of 18.3 nm was completely buried after the surrounding calcite face grew by only 12.3 nm (intercept of line at x-axis), demonstrating the micelle was contracted downward during its incorporation. This phenomenon was observed at other supersaturations as well, as shown in Fig. 3a, c of the main paper. (**f**) Schematic illustrating the method for obtaining the approximate true diameter of the micelle shown in **a**. (Also see Supplementary Note 2 for more detailed explanation.) (**g**) Resulting morphology of the micelle shown in **a** after burial based on the AFM measurements. As explained with Fig. 3f in the main paper, the initial width of a cavity surrounding the incorporated micelle corresponds closely to the diameter of the incorporated micelle. Therefore the micelle in **a** with an initial a height of 18.3 nm and an approximate true diameter of 89 nm upon its adsorption becomes an occlusion with a height of 12.3 nm and an approximate true diameter of 35 nm, giving a vertical contraction of about 33 % and a lateral compression of 61 %. All images were collected in contact mode.

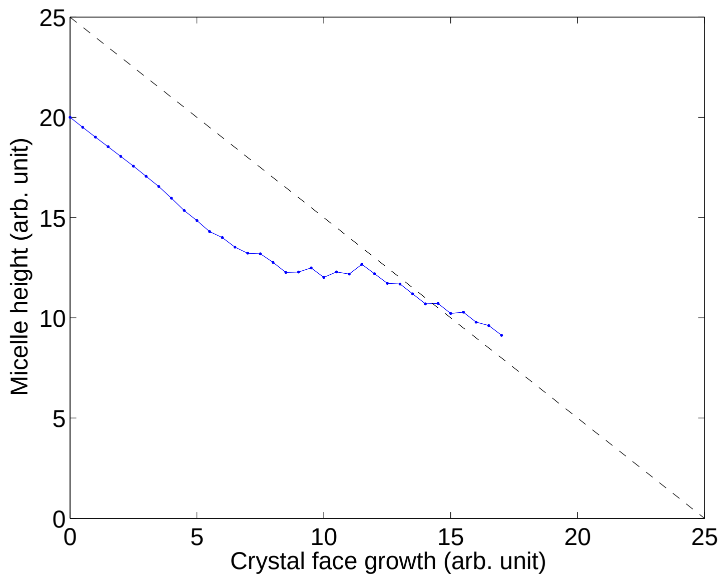

Supplementary Figure 6. Result of simulation of circular micelle incorporation into a crystal growing through layer-by-layer addition using the same parameters as in Fig. 4 of the main text. The relative height of the micelle over the crystal surface is plotted as a function of the height of the crystal surface. The dashed line corresponds to the condition where the micelle simply gets buried without any deformation

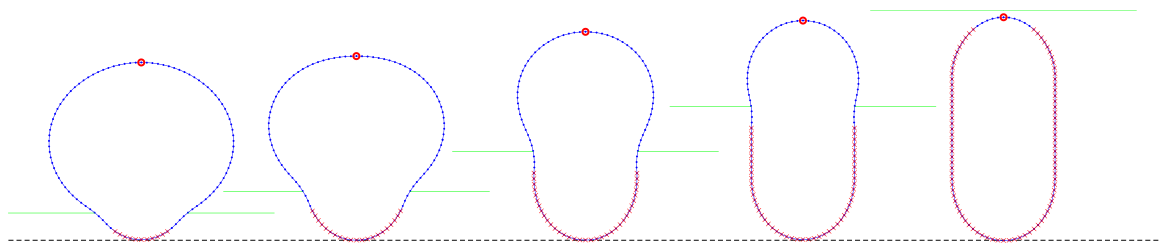

Supplementary Figure 7. Result of simulation of circular micelle incorporation into a crystal growing through layer-by-layer addition for K = 2, L_0 = 0.63, κ = 3, p = 10^{-4}. Micelle experiences lateral compression accompanied by downward contraction followed by upward extension during burial. The bottom dashed lines indicate the surface to which the micelle was initially attached, while the green solid lines represent the top surface of the crystal. The red crosses mark fixed portion of the (buried) micelle. The red circles mark the highest point of the micelle.

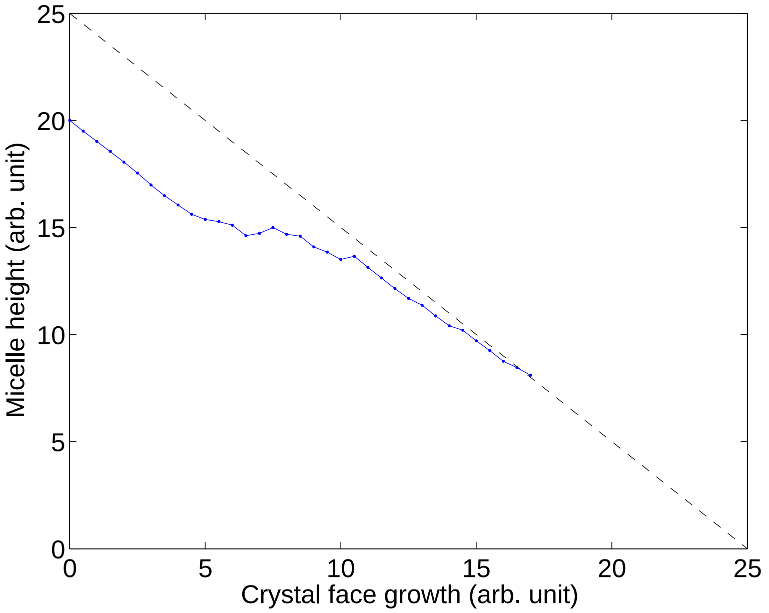

Supplementary Figure 8. Result of simulation of circular micelle incorporation into a crystal growing through layer-by-layer addition using the same parameters as those in Supplementary Fig. 7. The relative height of the micelle over the crystal surface plotted as a function of the height of the crystal surface. The dashed line corresponds to the condition where the micelle simply gets buried without any deformation.

Supplementary Note 1. Consideration of bulk free energy contribution in the physical model. We have also considered the effect of bulk free energy contribution, $F_{bulk} = -\int_{A_0}^{A} p(A)dA$, where $p(A) = \min\left[B\left(1-\frac{A}{A_0}\right), P_{max}\right]$ is the internal pressure of the micelle, A is the total area enclosed by the loop, B is the bulk modulus, and P_{max} is the maximum pressure in the micelle before solvent is expelled.

More explicitly,

$$p(A) = B\left(1-\frac{A}{A_0}\right) \quad \text{for } A > A_c \quad (1)$$

$$p(A) = P_{max} \quad \text{for } A \le A_c \quad (2)$$

$$F_{bulk}(A) = \frac{1}{2}\frac{B}{A_0}(A-A_0)^2 \quad \text{for } A > A_c \quad (3)$$

$$F_{bulk}(A) = -P_{max}(A-A_c) + \frac{1}{2}\frac{B}{A_0}(A_c-A_0)^2 \quad \text{for } A \le A_c \quad (4)$$

where

$$A_c = \left(1-\frac{P_{max}}{B}\right)A_0 \quad (5)$$

The functions $p(A)$ and $F_{bulk}(A)$ are illustrated below.

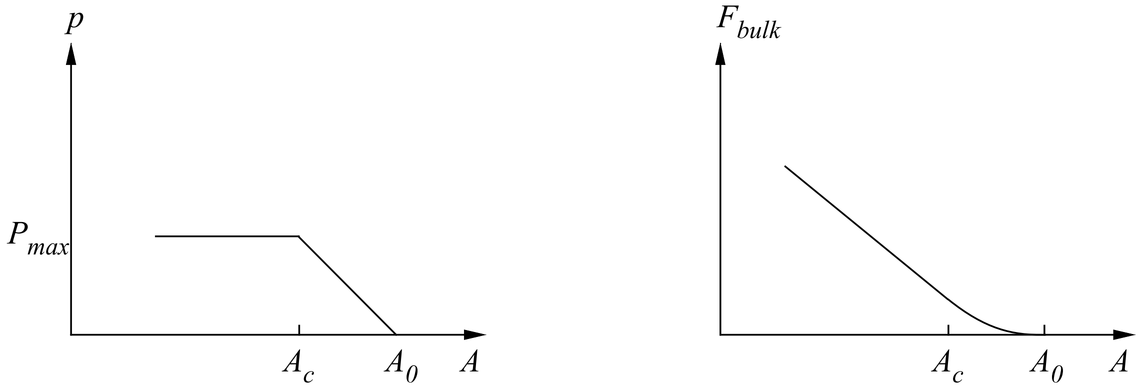

We have studied a wide range of values for B and P_{max} and found that if their values are too large the micelle shape remained essentially circular (i.e. incompressible), which would be inconsistent with our experimental observations. If B and P_{max} are sufficiently small, then the micelle deforms during burial, but the predicted shape evolution is qualitatively the same as that from the more simplified model in which both B and P_{max} are set to zero. Therefore, for simplicity, we omit the bulk contribution to the free energy in the results presented in the main text.

Supplementary Note 2. Method to obtain approximate true diameter of the micelle shown in Supplementary Fig. 5a. Unlike the measured heights of micelles, the diameters measured by AFM image are larger than the true diameters due to tip-sample convolution. The method used to calculate the true micelle diameter is illustrated by the following. Using an AFM tip with a nominal radius of 15 nm, a micelle that had just adsorbed to a step in Supplementary Fig. 5a was found to have an apparent diameter of 117 nm and a height of 18.3 nm. These values were then used to calculate the true diameter according to the schematic in Supplementary Fig. 5f. When the tip, moving from left to right, arrives at Location A, it first contacts the micelle at Location B, and when it arrives at Location C, it loses contact with the micelle at location D. Thus the distance between Location A and Location C is recorded as the apparent diameter of the micelle, but the true diameter is given by the distance from Location B to Location D. Taking the apparent diameter to be 117 nm and the true height to be 18.3 nm, a 15 nm tip gives a true diameter of 89 nm.

Supplementary Methods

Synthesis and characterization of sulfonated diblock copolymer SBA:PHPMA$_{30}$-PDPA$_{47}$. 2-(4-Morpholino) ethyl 2-bromoisobutyrate initiator (ME-Br; 280.0 mg) and 2-hydroxypropyl methacrylate monomer (HPMA, 4.32 g, target degree of polymerization, DP = 30) were dissolved in a solution of 2-propanol (IPA) and water (IPA/water = 95/5 % w/w) in a 100 mL two-neck round-bottomed flask to obtain a 50 % w/w solution of HPMA. This mixture was degassed using a stream of dry nitrogen gas for 1 h. Cu(I)Cl catalyst and 2,2'-bipyridine (bpy) (relative molar ratios of ME-Br: Cu(I)Cl: bpy = 1:1:2) were then added to the mixture quickly under a nitrogen blanket to start the polymerization (see Supplementary Fig. 2a). The reaction

mixture turned dark-brown and became viscous as the reaction proceeded. After approximately 2.5 h, the polymerization was essentially complete (more than 99 % conversion) as judged by ^{1}H NMR. A separately degassed 50 % w/w solution of 2-(diisopropylamino) ethyl methacrylate (DPA) in 95:5 % w/w IPA/water was then added to this reaction mixture using a syringe. The polymerization continued for 24 h and was then quenched by exposing the reaction mixture to air, followed by dilution with tetrahydrofuran (THF) (80 mL). The spent ATRP catalyst was removed by passing the green solution through a silica gel (0.063-0.2 μm particle diameter; Merck, Germany) column. The THF was partially removed under vacuum, and the crude block copolymer was precipitated into deionized water (500 mL). The recovered off-white copolymer was redissolved in THF and precipitated from water to remove traces of unreacted DPA monomer. A white solid was obtained after drying under vacuum. The overall yield of isolated, purified diblock copolymer precursor was 11.68 g (78 %). Gel permeation chromatography (GPC) analysis was performed using a refractive index detector with HPLC-grade THF eluent containing 2.0 % v/v triethylamine (TEA) at a flow rate of 1.0 mL min^{-1}. Toluene was used as an internal standard and the GPC column temperature was set at 30 ^{o}C. A series of near-monodisperse PMMA standards were used for calibration, which gave an M_n of 17,400 and an M_w/M_n of 1.21 for the PHPMA-PDPA diblock copolymer precursor. Assuming that the target DP of 30 was achieved for the first PHPMA block, comparison of the integrated signal (see Supplementary Fig. 2b) assigned to the protons (k, l) of the DPA residues at δ 2.8-3.3 with that of the oxyethylene protons (c, d, I, d') due to the HPMA and DPA residues at δ 4.0-4.5 indicated a mean diblock copolymer composition of $PHPMA_{30}$-$PDPA_{47}$. Then the $PHPMA_{30}$-$PDPA_{47}$ copolymer precursor (2.0 g) was dissolved in anhydrous THF (25 ml) in a 100 ml round-bottomed flask and two molar equivalents (relative to the hydroxyl groups of the copolymer) of 2-sulfobenzoic acid cyclic anhydride (SBA) and TEA were added to this solution under nitrogen. Esterification was allowed to proceed at 20 ^{o}C for 72 h (see Supplementary Fig. 2a). THF was then removed under vacuum, and the crude zwitterionic diblock copolymer was dissolved in saturated aqueous NaCl solution. The copolymer was purified by dialysis against saturated NaCl solution for at least two days to ensure complete removal of salt, followed by three days of dialysis against pure water and then freeze-drying overnight. ^{1}H NMR spectroscopy was used to assess the degree of esterification by comparing the two aromatic signals assigned to the sulfobenzoic acid groups (f, f') at δ 7.0 – 8.1 to the twelve methyl protons (m) due to the DPA residues at δ 1.2 (see Supplementary Fig. 2c). The final esterified diblock copolymer was obtained as an off-white solid and is denoted as 'SBA:$PHPMA_{30}$-$PDPA_{47}$'.

Supplementary References

1. Land, T. A., De Yoreo, J. J. & Lee, J. D. An in-situ AFM investigation of canavalin crystallization kinetics. *Surf. Sci.* **384,** 136–155 (1997).
